# Prospective observational study of young adult ischemic stroke patients

**DOI:** 10.1002/brb3.2283

**Published:** 2021-08-22

**Authors:** Renata Shihmanter, Joshua Friedman, Natali Kushner, Edward B. Miller, Ami Schattner

**Affiliations:** ^1^ Department of Medicine, Kaplan Medical Center Rehovot Israel; ^2^ Hebrew University Hadassah Medical School Jerusalem Israel

## Abstract

**Objective:**

Ischemic stroke (IS) in young patients may differ in etiology and prognosis from later‐life IS, which is much more common. A number of single‐center and population‐based cohorts of affected individuals have been published, but information on the long‐term prognosis of these patients is limited.

**Methods:**

IS patients (≤55 years), discharged over a 10‐year period, were evaluated and prospectively followed. Subgroups were evaluated for type of stroke, antecedent risk factors (RF), long‐term outcomes, and occupational status over several years of follow‐up.

**Results:**

178 IS individuals from 2001–2010 were divided into older (46–55, *n* = 118) and younger (18–45, *n* = 60) age groups. Traditional RF—hypertension, diabetes mellitus, hyperlipidemia—were significantly associated with IS, and increased with age. The distribution and type of IS were similar in both groups, except for an increase in small vessel IS among the older subgroup (*p* = .003).

Of the evaluable patients at 5.1 ± 2.5 years of follow‐up (*n* = 138), a similar proportion of patients in both subgroups had a recurrent IS, but no significant differences were found in most disability indices. Approximately one third of patients suffered from moderate to severe disability, and were unable to return to their prior work.

**Conclusions:**

Younger and older IS patients are generally predisposed from the same traditional RF which progress with age. Long‐term disabilities tend to worsen over time due to recurrent vascular events. These data emphasize the need for a strategy for early identification of the already well‐known stroke RF in younger stroke patients.

## Graphical abstract

Young (≤55 years old) ischemic stoke patients discharged over a 10 year period were prospectively evaluated and followed. Of 178 evaluable patients, raditional risk factors such as‐ hypertension, diabetes mellitus, hyperlipidemia were significantly associated with IS, and increased with age. The distribution and type of ischemic stroke were similar in both groups except for an increase in small vessel IS among the older subgroup (P = 0.003), and disability increased with the number of strokes.

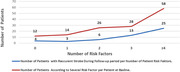

## LIMITATIONS

Our study has the following potential limitations: it included young IS cohorts from only two departments of our hospital. Full volume diagnostic studies were not completed by all patients. For statistical analysis, we did not separate patients with first and recurrent event on admission. Functional status on the day of discharge was not observed, and ethnic differences were not determined. The review of the follow‐up period, and the obtainment of information about a patient's functional or occupational status was done by telephone, and therapeutic follow‐up management was not included.

## 1 INTRODUCTION

The incidence of ischemic stroke (IS) has increased globally by 25% among adults under age 60 (Hankey, 2017; Virani et al., 2020). IS statistics have confirmed a trend of increased rates of stroke in all age groups in the 21st century all over the world. The incidence of IS doubles every 10 years starting from the age of 40 (Béjot et al., 2014; Hankey, 2017; Kissela et al., 2012; Krishnamurthi et al., 2013; Maaijwee et al., 2014; GBD 2016 Neurology Collaborators, 2019; Rozenthul‐Sorokin et al., 1996; Swerdel et al., 2016; Virani et al., 2020). The European Dijon Stroke Registry reported an increase of IS incidence for individuals under the age of 55, from 8.1 to 18.1 strokes per 100,000 patient‐years during two periods: 1985−1993 versus 2003−2011, respectively (Béjot et al., 2014). This indicated increasing hospitalization rates for both men and women aged 18–54 years, whose stroke risk factors (RF) doubled concurrently (Ferro et al., 2010; George et al., 2017; Krishnamurthi et al., 2013; Putaala et al., 2012). However, these patients are likely to differ from older IS patients in their RF, etiologic subgroups, and health outcomes (GBD 2016 Neurology Collaborators, 2019; Maaijwee et al., 2014). A number of single‐center and population‐based cohorts of IS in younger individuals have been published, but information on the long‐term prognosis of younger IS patients is still limited.

We therefore conducted a prospective study to evaluate and follow‐up adults younger than 55 years of age, following hospital discharge for acute IS. Analysis focused on risk factors (RF), etiology, stroke subtypes, and long‐term clinical outcomes. We tested the hypothesis that younger patients differ in their RF, IS characteristics, and outcomes, and aimed to compare IS patients from a younger subgroup (aged 18–45 years) with those from an older one (aged 46–55 years).

## 2 PATIENTS AND METHODS

All surviving patients aged 18–55 years, with a discharge diagnosis of IS between 2001 and 2010, from two departments of medicine out of four at an academic medical center, were invited for ambulatory evaluation and follow‐up. Older patients and individuals with active cancer, intra‐cerebral hemorrhage, or other neurological diseases, were excluded.

Chart review was done for each patient, and was complemented by a full medical history, physical examination, routine and ancillary diagnostic work‐up focusing on RF for IS. The latter included echocardiography, 24‐h ECG (Holter) monitoring, cervical artery Doppler study, and evaluation for hypercoagulability. RF were categorized as "traditional" (modifiable and non‐modifiable), and "additional" (less well‐documented or potentially modifiable) RF.

Neuroimaging was reviewed and evaluated for stroke subtypes according to the Trial of Org 10172 in Acute Stroke Treatment (TOAST) classification: large artery atherosclerosis, small vessel occlusion, cardioembolism, and other determined and undetermined etiologies. Arterial territory of IS was classified as hemispheric (including basal ganglia and thalamic regions), posterior fossa (including cerebellum and brain stem), multiple infarcts, and negative imaging.

Long‐term patient follow‐up included recurrent stroke and other cardiovascular events, epileptic seizures or death, and were based on information obtained from hospital charts, and contacting the patient's primary physician and/or death registries. Disabilities were evaluated through structured telephone interview with the patient (or caregiver when the patient was unable to communicate). The interview determined functional status by completing detailed and validated questionnaires, including the modified Rankin scale (mRS), measuring the degree of disability/dependence after stroke; the Barthel index (BI), reflecting current actual performance activities of daily living (ADL) and finally, the London Handicap Scale (LHS), providing a descriptive profile of disadvantages experienced in the domains of mobility, physical independence, orientation, social functioning, economic self‐sufficiency, and occupational status (OS).

The study was IRB approved. No therapeutic interventions were initiated by the authors. However, a new diagnosis with potential therapeutic implications was communicated to the patient's primary physician.

Statistical analysis was done using SAS 9.1.3 software (SPSS Inc.). Parameters of patients of different age groups (18–45 and 46–55) were compared by Pearson's *χ* ^2^ test and Fisher's Exact test to compare categorical variables across groups, as well as by Student's *t* test to compare means. Stepwise logistic regression model, using forward selection analysis, was used to determine the correlation between variables such as disability scores and age. *p* values < .05 were considered significant.

## 3 RESULTS

There were 202 eligible individuals who met the acceptance criteria for this study. Twenty‐four patients declined to participate. Of the 178 remaining participants, the mean age was 46.5 ± 7.6 years (median 48 years). Approximately one third of these individuals were 18–45 years old (60/178, 33.7%). This younger subgroup had a median age of 40 versus 51 in the older cohort (*n* = 118). Women constituted 54% of all study individuals, with gender distribution similar in the two subgroups. Intriguingly, 17/178 patients (10%) had a family history of stroke, and 83/178 (47%) reported a previous stroke/transient ischemic attack (TIA). These variables were not significantly different between the two cohorts (Table 1) and between genders (data not shown).

**TABLE 1:** Stroke risk factors classification by demographic age subgroups

	All (*n* = 178)	Age 18 to 45 years (*n* = 60)	Age 46 to 55 years (*n* = 118)	*p*
**Risk factors number per patient** *	3.1 ± 1.7	2.9 ± 1.8	3.5 ± 1.6	0.0003
**0–1**	31 (18)	20 (33)	11 (9)	0.0001
**2–3**	70 (39)	24 (40)	46 (39)	NS
**≥4 (range 4–8)** **	77 (43)	16 (27)	61 (52)	0.002
**Generally non‐modifiable risk factors**				
**Age, years (median)**	46.5 ± 7.6 (48)	38.1 ± 6.8 (40)	50.1 ± 2.8 (51)	NS
**Males**	82 (46)	25 (42)	58 (49)	NS
**Family history of stroke**	17 (10)	8 (13)	9 (8)	NS
**Previous stroke or transient ischemic attack, by history**	83 (47)	23 (38)	60 (51)	NS
**Well‐documented or modifiable risk factors**				
**Hypertension**	92 (52)	18 (30)	74 (63)	0.0001
**Tobacco smoking**	73 (41)	24 (40)	49 (42)	NS
**Obesity (BMI ≥ 30)**	32 (18)	6 (10)	26 (22)	NS
**Diabetes mellitus**	49 (28)	11 (18)	38 (32)	0.05
**Dyslipidemia**	107 (60)	20 (33)	87 (74)	0.0001
**Atrial fibrillation**	15 (8)	2 (3)	13 (11)	NS
**Coronary artery disease**	43 (24)	7 (12)	36 (31)	0.005
**Peripheral artery disease**	14 (8)	2 (3)	12 (10)	NS
**Other cardiac conditions** ^1^ ^#^	88 (49)	20 (33)	68 (58)	0.002
**Carotid artery stenosis**	17 (10)	4 (7)	13 (11)	NS
**Female hormonal factors**	12 (7)	10 (17)	2 (2)	0.003
**Less well‐documented or potentially modifiable risk factors**				
**History of migraine**	9 (5)	3 (5)	6 (5)	NS
**Alcohol abuse**	5 (3)	2 (3)	3 (3)	NS
**Obstructive sleep apnea syndrome**	7 (4)	0 (0)	7 (6)	NS
**Hyperhomocysteinemia**	7 (4)	2 (3)	5 (4)	NS
**Antiphospholipid syndrome**	26 (15)	13(22)	13 (11)	0.07
**Other hypercoagulable state** ^1^	12 (7)	5 (8)	7 (6)	NS
**Inflammation/Infection** ^1^ ^##^	41 (23)	11 (18)	30 (25)	NS

*Note*: Data are expressed as mean ± SD or *n* (%).

* Variables such as age and gender were not included.

** Age group 18–45: 4 risk factors in 8 patients, 5 in 6 patients, 7 and 8 in two patients; Age group 46–55: 4 risk factors in 32 patients, 5 in 19 patients, 6 in 8 patients, 7 and 8 in two patients.

^1^ One patient may have more than one risk factors.

^#^ Other cardiac conditions: heart failure/cardiomyopathy; valvular heart disease; cardiac arrhythmia (non‐atrial fibrillation) and others.

^##^ Autoimmune disease, Hepatitis C and others.

### 3.1 Risk factors

RF for study participants are presented in Table 1. Participants in the 46–55 age subgroup had significantly more RF per individual (*p* = .0003), and there were more subjects with four or more RF (*p* = .002) than in the younger subgroup. Traditional RF such as hypertension, diabetes, and dyslipidemia, as well as a history of previous atherosclerotic cardiovascular disease (ASCVD), were significantly more prevalent among individuals in the older versus the younger cohort (*p* = .002). In particular, coronary artery disease (CAD), atrial fibrillation (AF), carotid artery stenosis (CAS), and peripheral vascular disease (PVD), were all more common in the older subgroup, but only CAD reached a level of statistical significance.

Multivariate analysis showed a persistent strong correlation of IS in the older 46–55 age subgroup, with hypertension (OR = 3.9, 95% CI: 1.78–8.61, *p* = .002), dyslipidemia (OR = 2.9, 95% CI: 1.33–6.45, *p* < .0001), and ASCVD (OR = 3.15, 95% CI: 1.37–7.27, *p* = .04). In contrast, the younger 18–45 age cohort had a significantly lower prevalence of these traditional RF, but hormonal factors (16.7% vs. 1.7%, *p* = .003) and antiphospholipid syndrome (21.7% vs. 11%, *p* = .07) emerged as more prevalent in this group. Only a few patients had no identifiable RF; 12% in the younger, and 5% in the older subgroup, but this was not statistically significant.

Compared with females, male patients (82/178, 46%) had significantly more RF per individual (3.5 ± 1.8 vs. 2.8 ± 1.5, *p* = .002), with more diabetes (40% vs. 17%, *p* = .007), hypertension (62% vs. 43%, *p* = .001), smoking (51% vs. 32%, *p* = .01), and alcohol abuse (6% vs. 0%, *p* = .02). Men also had significantly more CAD (37% vs. 14%, *p* = .0004), CAS (15% vs. 5%, *p* = .04), and PVD (12% vs. 4%, *p* = .05) (Appendix 1). On multivariate analysis, the three most prominent RF in men remained diabetes (OR = 3.2, 95% CI: 1.42–7.41, *p* < .0001); ASCVD (OR = 4.1, 95% CI: 1.71–9.97, *p *= .001), and alcohol (*p* = .007). In contrast, women had more valvular heart diseases (16% vs. 4%, *p* = .02), and autoimmune diseases (19% vs. 2%, *p* = .004). As expected, hormonal factors in women retained their significance on multivariate analysis. Obesity, arrhythmia, and other RF showed no significant difference between genders.

### 3.2 Stroke subtypes

The relative proportion of types of IS as per TOAST classification is listed in Table 2. Only small vessel disease was significantly different between the cohorts, occurring more frequently in the older 46–55 age subgroup (45% vs. 22%, *p* = .003). Despite careful evaluation, the type of stroke could not be determined in 25% of younger, and 9% of older individuals (*p* = .007). No significant differences between genders were found, including on multivariate analysis (Appendix 1).

**TABLE 2:** Vascular territories of clinical symptoms, imaging features and etiology by TOAST classification of demographic age subgroups¹

	All (*n* = 178)	Age 18–45 years (*n* = 60)	Age 46–55 years (*n* = 118)	*p*
**Vascular territory by clinical symptoms**				
**Anterior circulation**	126 (71)	44 (73)	82 (70)	NS
**Posterior circulation**	25 (14)	7 (12)	18 (15)	NS
**Both**	27 (15)	9 (15)	18 (15)	NS
**Localization of ischemic lesions by neuroimaging** *				
**Cerebral hemispheres**	118 (66)	35 (58)	83 (70)	NS
**Posterior fossa localization**	35 (20)	8 (13)	27 (23)	NS
**Multiple infarcts**	65 (37)	21 (35)	44 (37)	NS
**Patients with negative imaging**	71 (40)	31 (52)	40 (34)	0.02
**Etiology by TOAST classification**				
**Large‐artery atherosclerosis**	14 (8)	4 (7)	10 (9)	NS
**Cardioembolism**	18 (10)	6 (10)	12 (10)	NS
**Small vessel disease**	66 (37)	13 (22)	53 (45)	0.003
**Other determined etiology**	36 (20)	17 (28)	19 (16)	NS
**Undetermined etiology**:	44 (25)	20 (33)	24 (20)	NS
Two or more causes identified	11 (6)	3 (5)	8 (6)	NS
Negative extensive evaluation	25 (14)	15 (25)	10 (9)	0.007
Incomplete evaluation	8 (5)	2 (3)	6 (5)	NS

*Note*: Data are expressed as *n* (%).

¹Percentages not equal to 100% because patients group with multiple infarcts may include more than one localization of visualized ischemic lesions with different localization, correlated with symptoms.

* Cerebral hemisphere (right or left); posterior fossa localization (cerebellum right or left and brain stem right or left); multiple infarcts (simultaneous multiple different brain infarcts) that appeared in one or different arterial territories, with same or different chronological age (per CT or MRI).

### 3.3 Arterial territory of stroke

According to clinical presentation or neuroimaging (cerebral hemispheres, posterior fossa or multiple infarcts) the vascular territories of the stroke were not significantly different between the two age subgroups (Table 2), or between male and female patients (Appendix 1). Negative neuroimaging studies ‐ occurred frequently in younger (52% vs. 34%, *p* = .02) and male (52% vs. 29%, *p* = .002). On multivariate analysis, the absence of hypertension (OR = .34, 95% CI: 0.17–0.7, *p* = .0001) or valvular disease (OR = 0.25, 95% CI: 0.08–0.77, *p* = .01) was found to be predictive of negative brain imaging.

### 3.4 Follow‐up

**FIGURE 1:**
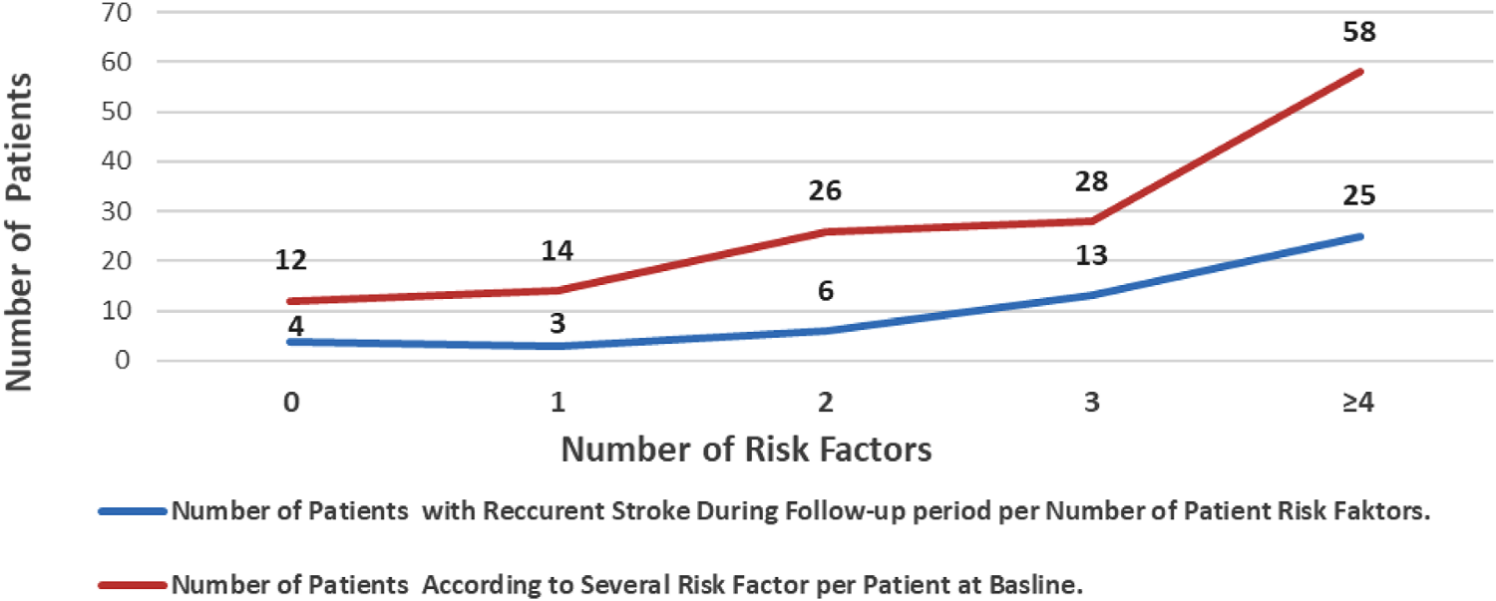
Recurrent stroke in the follow‐up period of 138 patients grouped on the basis of number of risk factors

Over the follow‐up period of 5.1 ± 2.5 years (similar in both age subgroups), 13/178 (7%) patients died; 3 (5%) in the younger and 10 (9%) in the older groups (*p* = NS) (Table 3). Cause of death was similarly distributed. Autoimmune disease (*p* = .02), alcohol abuse (*p* < .001), and older age were identified as potential predictors of mortality through multivariate analysis. As many as 32% of the patients had a recurrent stroke over the follow‐up period, but recurrence rate was not significantly different between the two age and gender subgroups, but was still correlated with the number of RF (Figure 1). When three or more RF were present, over 40% of the patients had recurrent IS— twice as many as patients with 1–2 RF, but this did not reach levels of statistical significance. Epileptic seizures were similarly distributed between the subgroups, occurring in about 8% of the patients. However, significantly more male versus female patients (20% vs. 9%, *p* = .05), and 46–55 vs. 18–45‐year‐old patients (20% vs. 7%, *p* = .002), had a recurrent cardiovascular event (Table 3, Appendix 1).

**TABLE 3:** Events during long‐term follow‐up period of 178 patients by age subgroups

	All *n* = 178	Age 18–45 years (*n* = 60)	Age 46–55 years (*n* = 118)	*p*
**Follow‐up (years)**	5.1 ± 2.4	5.2 ± 2.5	5.0 ± 2.3	NS
**Death**	13 (7)	3 (5)	10 (9)	NS
**Cause of death**:				
Stroke	1	0	1	NS
Congestive heart failure	2	1	1	NS
Myocardial infarct	4	1	3	NS
Sepsis	6	2	4	NS
**Recurrent stroke**	56 (32)	16 (27)	40 (34)	NS
**Recurrent cardiovascular event**	28 (16)	4 (7)	24 (20)	0.002
**Epileptic seizure**	14 (8)	4 (7)	10 (9)	NS

*Note*: Data are expressed as mean ± SD or *n* (%).

### 3.5 Functional status

Twenty‐seven patients (15%) were alive, but were lost to follow‐up or declined to respond despite their initial consent. Disability follow‐up was obtained in 138 patients, at a mean time interval of 5 years. These patients constituted approximately 80% of the original cohort in each of the age subgroups.

We used four different validated instruments (mRS, BI, LHS, and OS) to evaluate long‐term functional outcome and disability. Results are presented in Table 4 and Appendix 1. All instruments were consistent in showing that approximately 60% of the surviving IS patients emerged without disability, and were able to return to their previous employment. Both mRS and OS indicated that this positive outcome applied to significantly fewer patients in the 46–55 age subgroup, as compared to the younger 18–45 individuals (*p* ≤ .05). Indeed, this is reflected by the greater proportion of individuals in the older subgroup classified as "severe disability" (best seen in the 38% vs. 23% unable to work in the OS), but the difference did not reach statistical significance. Multivariate analysis identified four significant factors predictive of severe disability and inability to resume work: hypertension (OR = 0.37, 95% CI: 0.16–0.85, *p* = .007), recurrent stroke (by mRS) (OR = 3.54, 95% CI: 1.65–7.6, *p* = .0005), CAD (OR = 1.15, 95% CI: 0.04–0.55, *p* = .0009), and large artery disease (by TOAST classification) (OR = 0.08, 95% CI: 0.02–0.29, *p* = .0001). The same RF correlated with the inability to resume work in the gender subgroup as well (Appendix 1).

**TABLE 4:** Long‐term disability by modified Rankin scale, Barthel index, London handicap scale, and occupational status of 138 patients by age subgroups

	All (*n* = 138)	Age 18–45 years (*n* = 47)	Age 46–55 years (*n* = 91)	*p*
**Modified Rankin Scale**				
**No disability (score 0)**	80 (58)	33 (70)	47 (52)	0.05
**Minimal to moderate disability (score 1–3)**	42 (30)	10 (21)	32 (35)	NS
**Moderately severe to severe disability (score 4–5)**	16 (12)	4 (9)	12 (13)	NS
**Barthel index**				
**No to minimal disability (score 100–95)**	88 (64)	34 (72)	73 (80)	NS
**Mild to moderate disability (score 90–55)**	37 (27)	9 (19)	9 (10)	NS
**Severe disability (score <50)**	13 (9)	4 (9)	9 (10)	NS
**London handicap scale**				
**No disability (score 1)**	88 (64)	34 (72)	54 (60)	NS
**Minimal to moderate disability (score 0.9–0.55)**	4 (3)	0 (0)	4 (4)	NS
**Severe disability (score <0.5)**	46 (33)	13 (28)	33 (36)	NS
**Occupational status**				
**Return to work without changing occupation status**	82 (60)	34 (73)	48 (53)	0.03
**Return to work with changing occupation status**	10 (7)	2 (4)	8 (9)	NS
**Inability to work**	46 (33)	11 (23)	35 (38)	NS

*Note*: Data are expressed as *n* (%).

## 4 DISCUSSION

By definition, “stroke in the young” used to be an event occurring at or before age 44. However, today, due to the increasing span of life, and variability in the cut of upper age border (45–55 years) between statistic groups in the literature, the age period of "stroke in the young" is divided into “early adult life” before age 44, and "midlife” between ages 45 and 55 (Virani et al., 2020). So, our study, which took into account these two IS young groups for statistical analysis, has documented and brought for discussion, the following results.

### 4.1 Risk factors

Most individuals, who were males and in the 46–55 age group, were found to have multiple RF. Only a minority of the patients had one or no RF, and these individuals were significantly more prevalent in the younger subgroup. Modifiable RF (hypertension, dyslipidemia, DM, and associated ASCVD) increased with age and predominated. Most "classic" modifiable RF were already well represented in the younger (18–45 years) and male cohort; thus, comparisons of the age/gender subgroups were frequently found to lack statistical significance. The accumulation of traditional RF in males and with aging, as shown in a Danish group of 40,102 patients, may explain the male predominance among older patients as seen in the Helsinki study, but this proportion has been shown to equalize in patients over age 55 (Andersen et al., 2010; Putaala et al., 2009).

In contrast, younger individuals and females were more likely to have non‐traditional RF such as valvular heart disease (VHD), estrogen use, or an underlying autoimmune disease. This observation is consistent with other published studies (Andersen et al., 2010; Bushnell et al., 2014; Virani et al., 2020). In contrast to other investigators, we did not find any association with migraine headaches or atrial fibrillation in affected females (Bushnell et al., 2014; O'Donnell et al., 2016; Virani et al., 2020). Presence of the less well‐documented RF was not high in the age or gender groups, but their presence has significantly changed patient's follow‐up, with the increase of mortality in these groups, same as was reported in the Helsinki young stroke registry (Putaala et al., 2009).

On the whole, our findings are largely in agreement with previously published studies, and highlight the need for early and aggressive stroke prevention strategies in younger stroke patients (Aigner et al., 2017; Andersen et al., 2010; Putaala et al., 2009).

### 4.2 Stroke subtypes

Our data on stroke subtypes largely correlate with other published studies. For example, as in the Dijon Stroke Registry, one third of their cohort had "undetermined etiology and IS due to small vessel disease, cardioembolic episodes and large vessel disease increasing progressively with age" (Béjot et al., 2014). Comparison of our data together with other Israeli published data on young IS versus other countries, show a similar distribution of stroke subtypes, total and per age groups, without statistical significances (Appendix 2). This comparison is generalized and approximated because of differences between the studies using the classification system employed (TOAST vs. others), age group stratification, subtype gradation, and frequency of diagnostic tests utilized.

### 4.3 Territory of stroke

The topography of brain infarcts seen in our cohort is similar to that found in previously published studies, with predominance of anterior circulation lesions and no significant difference in either age or gender subgroups (Nedeltchev et al., 2005; Putaala et al., 2009). In contrast to previous reports, negative neuroimaging studies were frequently seen in our cohort, being significantly more common in the 18–45 subgroup. Most of these individuals lacked long‐term disability (by mRS) and classic vascular RF such as hypertension or VHD. These findings may be attributed to the general lack of sensitivity of neuroimaging studies to small vascular events. Negative neuroimaging studies have been reported in up to one third of patients with low IS severity, posterior circulation, or female gender (Doubal et al., 2011; Edlow et al., 2017). Neuroimaging with multiple infarcts in our cohort had a higher proportion (40%), similar to that seen in the Helsinki registry, but in contrast to the report by Kristensen et al. (1997) and Putaala et al. (2009).

### 4.4 Patient follow‐up

Over our follow‐up period of 5.1 ± 2.5 years, 13/178 (7%) patients died, the major cause of death being of vascular origin (54%). A recent long‐term follow‐up study in young IS patients demonstrated a 5‐year cumulative mortality ranging from 5.8% to 11%, 10‐year cumulative mortality from 12% to 17%, and 26.8% after 20 years. The cause of death in 55% of cases was of vascular origin, and in 28%, it was felt to be preventable (Bentur & Resnitzky, 2009; Kappelle et al., 1994; Leys et al., 2002; Naess et al., 2004; O'Donnell et al., 2016; Rutten‐Jacobs et al., 2013; Varona et al., 2004; Waje‐Andreassen et al., 2007). Our multivariate analysis of RF for the prediction of mortality found that older age and high consumption of alcohol were, as expected, closely correlated with death (*p* < .001). Surprisingly, however, we also found an association with autoimmune diseases (*p* = .02), which has not been previously reported (Waje‐Andreassen et al., 2007).

Post‐stroke epilepsy was demonstrated in 8% of our young IS cohort, similar to the 2.4−14.4% reported rates in other studies (Leys et al., 2002; Naess et al., 2004; Spengos & Vemmos, 2010; Varona et al., 2004 ). The risk of recurrent cardiovascular event following a stroke is known to be elevated with the time, with a calculated cumulative risk of 17%, while the cumulative risk for recurrent stroke is 20% or more (Naess et al., 2004; Rutten‐Jacobs et al., 2013; Spengos & Vemmos, 2010; Varona et al., 2004 ). Recurrent cardiovascular events in our study population were significantly more frequent in males and older patients. Approximately one third of our cohort had a recurrent IS during the follow‐up period, and this frequency was not significantly different between the age and gender subgroups. The published data on long‐term risk, and predictors of recurrent ischemic events after IS, suggest an association with the number of modifiable age‐specific RF, the severity of the previous event, and the level of aggressive intervention in RF modification (García et al., 2016; Moerch‐Rasmussen et al., 2016; Pezzini et al., 2014; Rutten‐Jacobs et al., 2013). In our small cohort, we did not find that the number of RF per patient was a statistically significant or predictable factor for recurrent IS during the follow‐up period. This may be because cases of recurrent IS in the follow‐up period are mostly dependent on the therapeutic management of RF, which was not included in our study.

### 4.5 Long‐term disability

The sub‐analysis of the Global Burden of Disease (GBD) study of 2019 showed stroke as the top‐ranked age‐related cause of disability‐adjusted life‐years (DALYs) worldwide (GBD 2019 Diseases & Injuries Collaborators, 2020). Proportions of individuals with poor functional outcome (mRS score > 2) among young stroke survivors range from 6% to 20% after a mean follow‐up of 3−12 years (Arntz et al., 2013; Leys et al., 2002; Naess et al., 2004; Synhaeve, Arntz, et al., 2014; Synhaeve, Schaapsmeerders, et al., 2015; Varona et al., 2004). Seventy percent (*p* = .05) of our young patients, after IS, recovered by mRS and BI without disability. Moderate‐to‐severe disability was the same in the gender and age groups. We did not check the level of disability of the patients on the day of discharge, but other Israeli studies found no significant differences between the younger and older patients by mRS (Lutski et al., 2017; Telman et al., 2008).

IS in the young leads to limitations in the quality of life and occupational status.

A third of all our patients remain severely disabled and are thus unable to return to work. These poor outcomes were found somewhat less likely in the 18–45 subgroup (23% vs. 38%), but did not correlate with gender, and were comparable to the results reported by Varona et al. (2004). Our multivariate analysis identified four significant factors predictive of severe disability and the inability to resume work: large artery disease by TOAST classification, hypertension, recurrent stroke, and CAD. These results were identical to other published studies (Leys et al., 2002; Naess et al., 2004; Varona et al., 2004). Other newly reported measures of severe disability in young IS patients have been shown to correlate with long‐term disability, and subsequent late inability to return to work, but were not evaluated sufficiently in our cohort. These include post‐stroke fatigue, cognitive severity, depression, and sexual dysfunction as poor functional outcomes (Kauranen et al., 2013; Maaijwee, Arntz, et al., 2015; Maaijwee, Rutten‐Jacobs, et al., 2014).

## 5 CONCLUSION

Younger patients with IS are predisposed by the same traditional vascular stroke RF as older individuals, and these progressively increase with age. We found no significant age‐ and gender‐related differences in terms of RF, etiologies, stroke subtype, and long‐term clinical outcome. The risk of long‐term disability worsens with time due to recurrent vascular events. These data emphasize the need for a strategy for early identification of the already well‐known stroke RF, and to institute an aggressive therapeutic management in all patient groups as a primary and secondary prevention of recurrent stroke.

## Supporting information

Supporting information

Supporting information
