# Supplementary material for: Prospective observational study of young adult ischemic stroke patients
Source: Brain Behav. 2021 Aug 22;11(9):e2283. doi: 10.1002/brb3.2283 (PMC8442588; doi:10.1002/brb3.2283)
Supplement: Supplementary file 1 — Supporting information [file BRB3-11-e2283-s002.doc]

**Appendix1: Tables of Statistics Study Data of Gender Subgroups Analysis.**

**Table I. Stroke Risk Factors Classification by Demographic Gender Subgroups**

| **p** | **Females**  **(n=96)** | **Males**  **(n=82)** | **All**  **(n=178)** |  |
| --- | --- | --- | --- | --- |
| 0.002 | 2.8 ± 1.5 | 3.5±1.8 | 3.1±1.7 | **Risk factors number per patient*** |
| NS | 20 (21) | 11 (13) | 31(18) | **0-1** |
| NS | 43(45) | 27(33) | 70(39) | **2-3** |
| 0.01 | 33(34) | 44(54) | 77(43) | **≥4 (range 4-8)**** |
| **Generally Non-Modifiable Risk Factors** | | | | |
| 0.07 | 45.6±7.8 | 47.6±7.2 | 46.5±7.6  (48) | **Age, years**  **(median)** |
| NS | 12(13) | 5 (6) | 17 (10) | **Family history of stroke** |
| NS | 48(50) | 35(43) | 83(47) | **Previous stroke or transient ischemic attack, by history** |
| **Well-Documented or Modifiable Risk Factors** | | | | |
| 0.01 | 41(43) | 51(62) | 92(52) | **Hypertension** |
| 0.01 | 31(32) | 42(51) | 73(41) | **Tobacco smoking** |
| NS | 21(22) | 11(13) | 32(18) | **Obesity** (BMI ≥ 30) |
| 0.0007 | 16(17) | 33(40) | 49(28) | **Diabetes mellitus** |
| NS | 59(62) | 48(59) | 107(60) | **Dyslipidemia** |
| NS | 8(8) | 7(9) | 15(8) | **Atrial fibrillation** |
| 0.0004 | 13(14) | 30(37) | 43(24) | **Coronary artery disease** |
| 0.06 | 4(4) | 10(12) | (8)14 | **Peripheral artery disease** |
| 0.001 | 36(38) | 52(63) | 88(49) | **Other cardiac conditions** ^1^# |
| 0.04 | 5(5) | 12(15) | 17(10) | **Carotid artery stenosis** |
| 0.003 | 12(13) | 0 | 12(7) | **Female hormonal factors** |
| **Less Well-Documented or Potentially Modifiable Risk Factors** | | | | |
| NS | 6(6) | 3(4) | 9(5) | **History of migraine** |
| 0.02 | 0(0) | 5(6) | 5(3) | **Alcohol abuse** |
| NS | 3(3) | (5) 4 | 7(4) | **Obstructive sleep apnea syndrome** |
| NS | 4(4) | 3(4) | 7(4) | **Hyperhomocysteinemia** |
| NS | 13(14) | 13(16) | 26(15) | **Antiphospholipid syndrome** |
| NS | 7(7) | 5(6) | 12(7) | **Other hypercoagulable state¹** |
| 0.007 | 30(31) | 11(13) | 41(23) | **Inflammation/Infection** ¹## |

Data are expressed as mean ± SD or n (%);

* Variables such as age and gender were not included

** Male: 4 risk factors in 19 patients, 5 in 17 patients, 6 in 4 patients, 7 in 2 patients and 8 in 2 patients;

Female: 4 risk factors in 21 patients, 5 in 8 patients, 6 in 4 patients;

¹ One patient may have more than risk factors

# Other cardiac conditions: Heart failure/Cardiomyopathy; Valvular heart disease; Cardiac arrhythmia (non-atrial fibrillation) and others)

## Autoimmune disease, Hepatitis C and others

**Table 2. Vascular Territories of Clinical Symptoms, Imaging Features and Etiology by TOAST Classification of Demographic Gender Subgroups¹.**

| **p** | **Females**  **(n=96)** | **Males**  **(n=82)** | **All**  **(n=178)** |  |
| --- | --- | --- | --- | --- |
| **Vascular Territory by Clinical Symptoms** | | | | |
| NS | 73(76) | 53(65) | 126(71) | **Anterior circulation** |
| NS | 11(11) | 14(17) | 25(14) | **Posterior circulation** |
| NS | 12(13) | 15(18) | 27(15) | **Both** |
| **Localization of Ischemic Lesions by Neuroimaging*** | | | | |
| NS | 61(64) | 57(70) | 118(66) | **Cerebral hemispheres** |
| NS | 15(16) | 20(24) | 35(20) | **Posterior fossa localization** |
| NS | 34(35) | 31(38) | 65(37) | **Multiple infarcts** |
| 0.002 | 28(29) | 43(52) | 71(40) | **Patients with negative imaging** |
| **Etiology by TOAST classification** | | | | |
| NS | 6(6) | 8(10) | 14(8) | **Large-artery atherosclerosis** |
| NS | 10(10) | 8(10) | 18(10) | **Cardioembolism** |
| NS | 32(33) | 34(41) | 66(37) | **Small-vessel disease** |
| NS | 19(20) | 17(21) | 36(20) | **Other determined etiology** |
| NS | 29(30) | 15(18) | 44(25) | **Undetermined Etiology:** |
| NS | 7(7) | 4(5) | 11(6) | Two or more causes identified |
| NS | 18(19) | 7(8) | 25(14) | Negative extensive evaluation |
| NS | 4(4) | 4(5) | 8(5) | Incomplete evaluation |

Data are expressed as n (%).

¹ Percentages not equal to 100% because patients group with multiple infarcts may include more than one localization of visualized ischemic lesions with different localization, correlated with symptoms.

***** Cerebral hemisphere (Right or Left); Posterior Fossa localization (Cerebellum Right or Left and Brain stem Right or Left); Multiple infarcts (Simultaneous multiple different brain infarcts' that appeared in one or different arterial territories, with same or different chronological age (per CT or MRI).

**Table 3. Events During Long-Term Follow-up Period of 178 Patients by Gender Subgroups.**

| **p** | **Females**  **(n=96)** | **Males**  **(n=82)** | **All**  **n=178** |  |
| --- | --- | --- | --- | --- |
| NS | 5.0±2.3 | 5.2±2.5 | 5.1±2.4 | **Follow-up (years)** |
| NS | 4(4) | 9(11) | 13(7) | **Death** |
|  | | | | **Cause of death:** |
| NS | 0 | 1 | 1 | Stroke  Congestive heart failure  Myocardial infarct  Sepsis |
| NS | 1 | 1 | 2 |
| NS | 1 | 3 | 4 |
| NS | 2 | 4 | 6 |
| NS | 28(29) | 28(34) | 56(31) | **Recurrent stroke** |
| 0.05 | 9(9) | 16(20) | 28(16) | **Recurrent cardiovascular event** |
| NS | 6(6) | 8(10) | 14(8) | **Epileptic seizure** |

Data are expressed as n (%);

**Table 4. Long-Term Disability by Modified Rankin Scale, Barthel Index, London Handicap Scale and Occupational Status of 138 Patients by Gender Subgroups.**

| **p** | **Females**  **(n=76)** | **Males**  **(n=62)** | **All**  **(n=138)** |  |
| --- | --- | --- | --- | --- |
| **Modified Rankin Scale** | | | | |
| NS | 47(62) | 33(53) | 80(58) | **No disability**  **(score 0)** |
| NS | 20(26) | 22(36) | 42(30) | **Minimal to moderate disability**  **(score 1-3)** |
| NS | 9(12) | 7(11) | 16(12) | **Moderately severe to severe disability**  **(score 4-5)** |
| **Barthel Index** | | | | |
| NS | 52(69) | 36(58) | 88(64) | **No to minimal disability**  **(score 100-95)** |
| NS | 17(22) | 22(35) | 37(27) | **Mild to moderate disability**  **(score 90-55)** |
| NS | 7(9) | 4(7) | 13(9) | **Severe disability**  **(score < 50)** |
| **London Handicap Scale** | | | | |
| NS | 52(68) | 36(58) | 88(64) | **No disability**  **(score 1)** |
| NS | 3(4) | 1(2) | 4(3) | **Minimal to moderate disability**  **(score 0.9-0.55)** |
| NS | 21(28) | 25(40) | 46(33) | **Severe disability**  **(score <0.5)** |
| **Occupational Status** | | | | |
| NS | 47(62) | 35(57) | 82(60) | **Return to work without changing occupation status** |
| NS | 5(7) | 5(8) | 10(7) | **Return to work with changing occupation status** |
| NS | 24(31) | 22(35) | 46(33) | **Inability to work** |

Data are expressed n (%);
