# Supplementary material for: Prospective observational study of young adult ischemic stroke patients
Source: Brain Behav. 2021 Aug 22;11(9):e2283. doi: 10.1002/brb3.2283 (PMC8442588; doi:10.1002/brb3.2283)
Supplement: Supplementary file 2 — Supporting information [file BRB3-11-e2283-s001.doc]

**Appendix 2.**

**Distribution of Stroke Etiology by TOAST Classification in Different Countries and Israel.**

**I.**

**
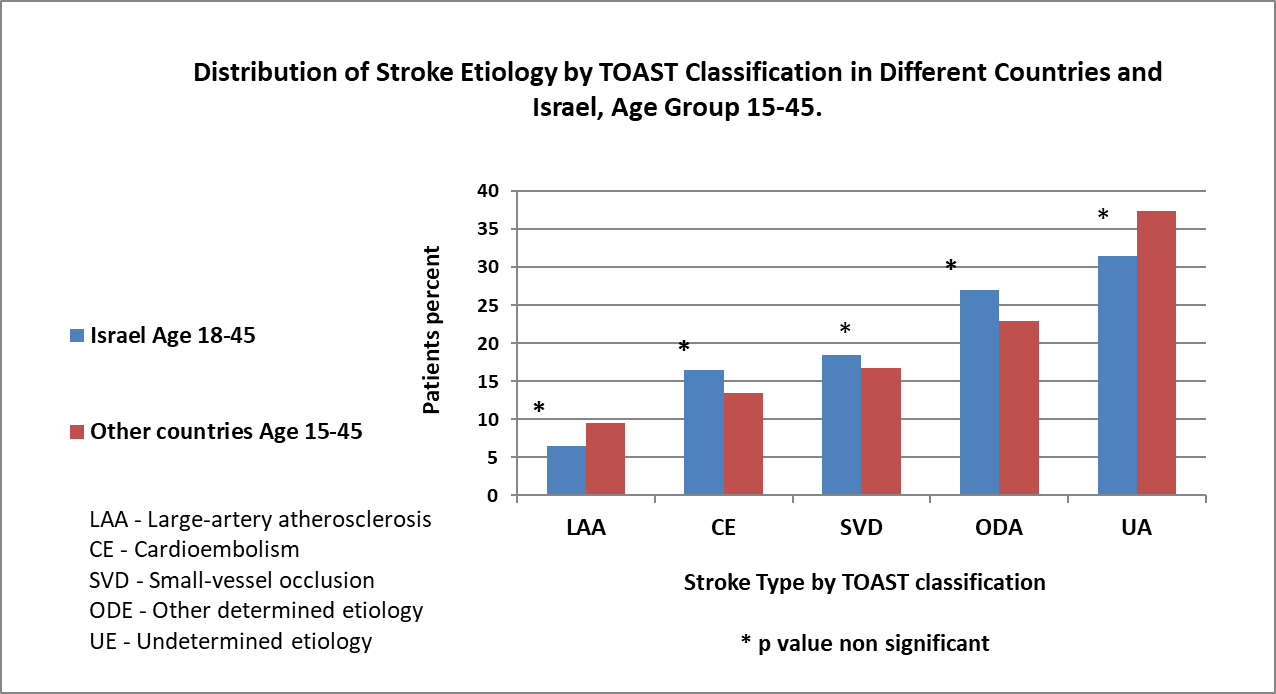
**

**II.**

**
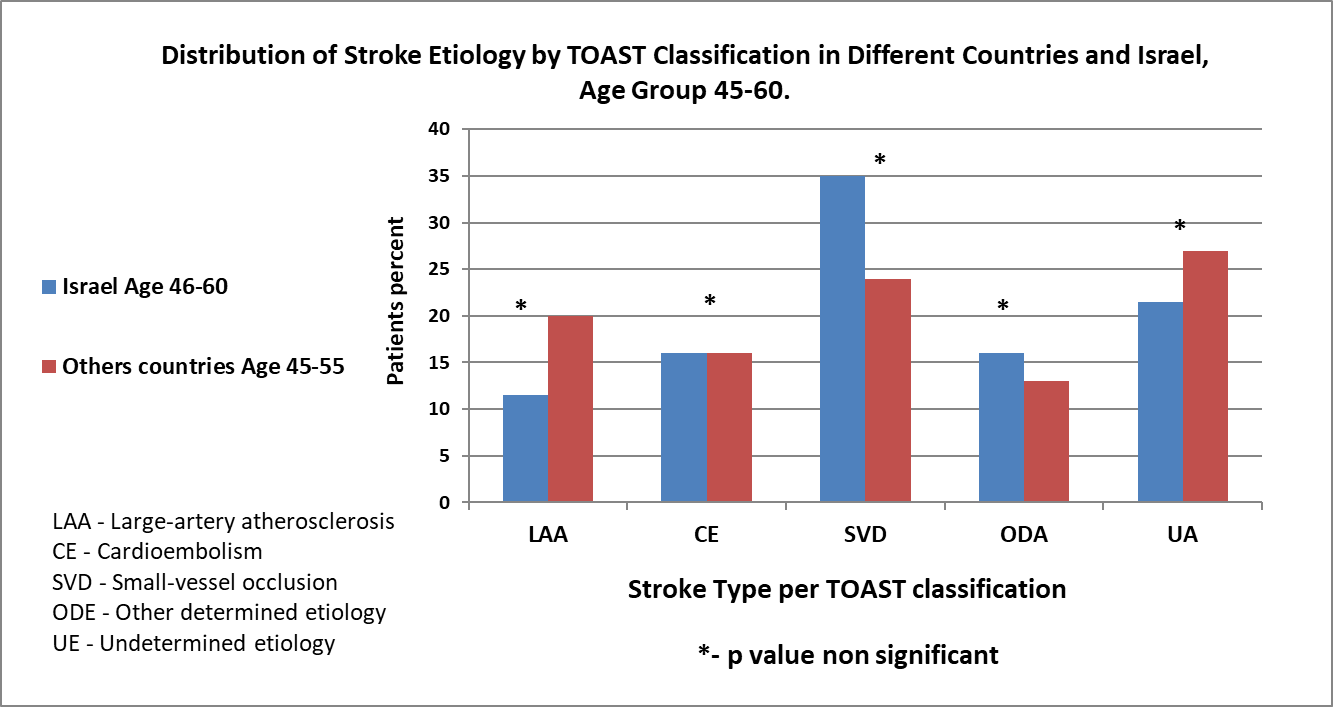
**

1. **Distribution of Stroke Etiology by TOAST Classification in Different Countries and Israel, Age Group 15-45**

| **UE**  **(%)** | **ODA**  **(%)** | **SVD**  **(%)** | **CE**  **(%)** | LAA  (%) | **Age** | **Number of patients** | **Country** | **Years of study** | **Year of publication** | **Authors** |
| --- | --- | --- | --- | --- | --- | --- | --- | --- | --- | --- |
| **Other Countries** | | | | | | | | | | |
| 44 | 28 | 14 | 8 | 6 | 15-45 | 356 | Canada | 1993-1997 | 2000 | Chan et al |
| 37 | 30 | 7 | 22 | 4 | 15-44 | 544 | Finland | 1994-2007 | 2009 | Putaala et al |
| 53 | 20 | 4 | 15 | 8 | 16-44 | 131 | France | 2006-2010 | 2011 | Larrue et al |
| 62 | 22 | 5 | 2 | 9 | 15-45 | 287 | France | 1992-1996 | 2002 | Leys et al |
| 35 | 21 | 7 | 20 | 17 | <45 | 285 | Germany | 1998-1999 | 2001 | Grau et al |
| 45 | 16 | 8 | 31 | 0 | 15-44 | 13 | Germany | 1994-1998 | 2001 | Kolominsky-Rabas PL |
| 28 | 8 | 54 | 3 | 7 | 15-45 | 124 | Iran | 2000-2005 | 2006 | Ghandehari et all |
| 27 | 22 | 32 | 6 | 13 | 25-45 | 88 | Korea | 2003-2009 | 2011 | Lee et al |
| 17 | 27 | 17 | 18 | 21 | 15-44 | 149 | Korea | 1994-1997 | 2000 | Kwon et al |
| 36 | 22 | 18 | 5 | 19 | 15-45 | 272 | Spain | 1974-2002 | 2007 | Verona et al |
| 33 | 30 | 24 | 9 | 4 | 16-45 | 203 | Swetzerland | 1997-2000 | 2005 | Nedeltchev et al |
| 50 | 20 | 7 | 16 | 7 | 16-45 | 284 | Switzerland | 2008-2012 | 2015 | Simonetti et al |
| 25 | 25 | 22 | 20 | 8 | 15-45 | 241 | Taiwan | 1997-2001 | 2002 | Lee et al |
| 32 | 32 | 15 | 13 | 8 | 15-44 | 428 | US | 1988-1991 | 1998 | Kittner et al |
| **37.4** | **23** | **16.7** | **13.4** | 9.5 | **Average** | **3405** | **Sum** |  | | |
| **Israel** | | | | | | | | | | |
| 30 | 26 | 23 | 15 | 6 | 18-45 | 87 | Israel | 2000-2007 | 2007 | Telman et al |
| 31 | 28 | 10 | 22 | 10 | 18-45 | 60 | Israel | 2001-2009 | - | Present study |
| **31.5** | **27** | **18.5** | **16.5** | 6.5 | **Average** | **147** | **Sum** |  | | |
| **NS** | **NS** | **NS** | **NS** | **NS** | **NS** | **p** | **Other countries / Israel** | | | |

LAA - Large-artery atherosclerosis; CE – Cardioembolism; SVD - Small-vessel disease;

ODE - Other determined etiologies; UE - Undetermined Etiology

1. **Distribution of Stroke Etiology by TOAST Classification in Different Countries and Israel, Age Group 45-60.**

| **UE**  **(%)** | **ODA**  **(%)** | **SVD**  **(%)** | **CE**  **(%)** | LAA  (%) | **Age** | **Number patients** | **Country** | **Years studied** | **Year published** | **Authors** |
| --- | --- | --- | --- | --- | --- | --- | --- | --- | --- | --- |
| **Other countries** | | | | | | | | | | |
| 27 | 21 | 21 | 17 | 12 | 45-49 | 464 | Finland | 1994-2007 | 2009 | Putaala et al |
| 39 | 10 | 12 | 19 | 20 | 45-54 | 187 | France | 2006-2010 | 2011 | Larrue et al |
| 14 | 3 | 33 | 20 | 30 | 45-54 | 30 | Germany | 1994-1998 | 2001 | Kolominsky-Rabas PL |
| 23 | 10 | 40 | 12 | 15 | 45-49 | 103 | Korea | 2003-2009 | 2011 | Lee et al |
| 34 | 20 | 13 | 13 | 20 | 46-55 | 340 | Switzerland | 2008-2012 | 2015 | Simonetti et al |
| 27 | 13 | 24 | 16 | 20 | **Average** | 1124 | **Sum** |  | | |
| **Israel** | | | | | | | | | | |
| 23 | 16 | 25 | 22 | 14 | 46-60 | 347 | Israel | 2000-2007 | 2007 | Telman et al |
| 20 | 16 | 45 | 10 | 9 | 46-55 | 118 | Israel | 2001-2009 | - | Present study |
| 21.5 | 16 | 35 | 16 | 11.5 | **Average** | 465 | **Sum** |  | | |
| **NS** | **NS** | **NS** | **NS** | **NS** | **NS** | **p** | **Other countries / Israel** | | | |

LAA - Large-artery atherosclerosis; CE – Cardioembolism; SVD - Small-vessel disease;

ODE - Other determined etiologies; UE - Undetermined Etiology

**References**

| Cerrato P, Grasso M, Imperiale D, Priano L, Baima C, Giraudo M, Rizzuto A, Azzaro C, Lentini A, Bergamasco B. Stroke in Young Patients: Etiopathogenesis and Risk Factors in Different Age Classes. Cerebrovasc Dis 2004;18:154–159 |
| --- |
| Chan MT, Nadareishvili ZG, Norris JW. Diagnosticstrategies in young patients with ischemic stroke incanada. Can J Neurol Sci 2000; 27:120-4. |
| Deleu D, Inshasi J, Akhtar N, Ali J, Vurgese T, Ali S, Rajan M, AlMutairy M, Zayed A, Paulose G, Nouri K, Thussu A, Miyares FR, Abdeen T, AlHail H, Alshubaili A, Mahmoud H. Risk factors, management and outcome of subtypes of ischemic stroke: a stroke registry from the Arabian Gulf. J Neurol Sci 2011 January 15;300(1-2):142-7 |
| Dharmasaroja PA, Muengtaweepongsa S, Lechawanich C, Pattaraarchachai J. Causes of Ischemic Stroke in Young Adults in Thailand A Pilot Study. J Stroke Cerebrovasc Dis. 2011;20(3):247-50 |
| Fromm A , Waje-Andreassen U, Thomassen L, Naess H . Comparison between Ischemic Stroke Patients <50 Years and ≥50 Years Admitted to a Single Centre: The Bergen Stroke Study. Stroke Res Treat. 2011 Jan 20;2011:183256. |
| Ghandehari K, Izadi Moud Z. Incidence and etiology of ischemic stroke in Persian young adults. Acta Neurol Scand 2006: 113: 121–124. |
| Grau AJ, Weimar C, Buggle F, et al. Risk factors, outcome and treatment in subtypes of ischemic stroke. Stroke. 2001;32:2559-2566 |
| Han SW, Kim SH, Lee JY, Chu CK, Yang JH, Shin HY, Nam HS, Lee BI, Heo JH. A new subtype classification of ischemic stroke based on treatment and etiologic mechanism. Eur Neurol. 2007;57(2):96-102. |
| Henrotin JB, Besancenot JP, Bejot Y, Giroud M. Short-term effects of ozone air pollution on ischaemic stroke occurrence: a case-crossover analysis from a 10-year population-based study in Dijon, France. Occup Environ Med 2007 July;64(7):439-45. |
| Kimura K, Kazui S, Minematsu K, Yamaguchi T. Japan Multicenter Stroke Investigators’ Collaboration (J-MUSIC). Hospital-based prospective registration of acute ischemic stroke and transient ischemic attack in Japan. J Stroke Cerebrovasc Dis. 2004 January–February; 20(1): 1-11. |
| Kittner SJ, Stern BJ,Wozniak M, et al. Cerebral infarction in young adults: The Baltimore-Washington Cooperative Young Stroke Study. Neurology 1998;50:890–894 |
| Koch S, Pabon D, Rabinstein AA, Chirinos J, Romano JG, Forteza A. Stroke etiology among Haitians living in Miami. Neuroepidemiology 2005;25(4):192-5 |
| Kolominsky-Rabas PL, Weber M, Gefeller O, Neundoerfer B, Heuschmann PU. Epidemiology of ischemic stroke subtypes according to TOAST criteria: incidence, recurrence, and long-term survival in ischemic stroke subtypes: a population-based study. Stroke 2001 December 1;32(12):2735-40. |
| Kwon SU, Kim JS, Lee JH, Lee MC (2000) Ischemic stroke in Korean young adults. Acta Neurol Scand 13(1):146–152 |
| larrue V, Berhoune N, Massabuau , Calviere L, Raposo N, Viguier , asr . Etiologic investigation of ischemic stroke in young adults. Neurology 2011;76:1983–1988 |
| larrue V, Berhoune N, Massabuau , Calviere L, Raposo N, Viguier , asr . Etiologic investigation of ischemic stroke in young adults. Neurology 2011;76:1983–1988 |
| Lavados PM, Sacks C, Prina L, Escobar A, Tossi C, Araya F, Feuerhake W, Galvez M, Salinas R, Alvarez G. Incidence, case-fatality rate, and prognosis of ischaemic stroke subtypes in a predominantly Hispanic-Mestizo population in Iquique, Chile (PISCIS project): a community-based incidence study. Lancet Neurol 2007 February;6(2):140-8. |
| Lee D, Heo SH, Kim HD, Chang D. Stroke subtypes and risk factors of ischemic stroke in young Korean adults. Neurology Asia 2011; 16(4) : 281 – 289 |
| Lee TH, Hsu WC, Chen CJ, et al. Etiologic studyof young ischemic stroke in Taiwan. Stroke 2002;33:1950-5. |
| Leys D, Bandu L, Hénon H, et al. Clinical outcome in 287 consecutive young adults (15 to 45 years) with ischemic stroke. Neurology 2002; 59: 26–33. |
| Low molecular weight heparinoid, ORG 10172 (danaparoid), and outcome after acute ischemic stroke: a randomized controlled trial. The Publications Committee for the Trial of ORG 10172 in Acute Stroke Treatment (TOAST) Investigators. JAMA 1998 April 22;279(16):1265-72. |
| Naess H, Idicula T, Brogger J, Waje-Andreassen U, Thomassen L. High proportion of lacunar strokes at night: the Bergen stroke study. J Stroke Cerebrovasc Dis 2011 September;20(5):424-8. |
| Naess H, Nyland HI, Thomassen L, Aarseth J, Myhr KM (2004) Etiology of and risk factors for cerebral infarction in young adults in western Norway: a population-based case-control study. Eur J Neurol 11:25–30 |
| Nedeltchev K, der Maur TA, Georgiadis D, Arnold M, Caso V,Mattle HP, Schroth G, Remonda L, Sturzenegger M, Fischer U,Baumgartner RW (2005) Ischaemic stroke in young adults: predictors of outcome and recurrence. J Neurol Neurosurg Psychiatr 76:191–195 |
| Palm F, Urbanek C, Wolf J, Buggle F, Kleemann T, Hennerici MG, Inselmann G, Hagar M, Safer A, Becher H, Grau AJ.Etiology, Risk Factors and Sex Differences in Ischemic Stroke in the Ludwigshafen Stroke Study, a Population-Based Stroke Registry. Cerebrovasc Dis 2012;33:69-75 |
| Poppert H, Sadikovic S, Sander K, Wolf O, Sander D. Embolic signals in unselected stroke patients: prevalence and diagnostic benefit. Stroke 2006 August;37(8):2039-43. |
| Putaala J, Metso AJ, Metso TM, Konkola N, Kraemer Y,Haapaniemi E, Kaste M, Tatlisumak T (2009) Analysis of 1008 consecutive patients aged 15 to 49 with first-ever ischemic stroke:the Helsinki young stroke registry. Stroke 40:1195–1203 |
| Putaala J, Metso AJ, Metso TM, Konkola N, Kraemer Y,Haapaniemi E, Kaste M, Tatlisumak T (2009) Analysis of 1008 consecutive patients aged 15 to 49 with first-ever ischemic stroke:the Helsinki young stroke registry. Stroke 40:1195–1203 |
| Rasura M, Spalloni A, Ferrari M, De Castro S, Patella R, Lisi F, Beccia M. A case series of young stroke in Rome. Eur J Neurol 2006; 13:146–152 |
| Rodriguez GJ, Cordina SM, Vazquez G, Suri MF, Kirmani JF, Ezzeddine MA, Qureshi AI. The hydration influence on the risk of stroke (THIRST) study. Neurocrit Care 2009;10(2):187-94. |
| Roquer J, Campello AR, Gomis M. Sex differences in first-ever acute stroke. Stroke 2003 July;34(7):1581-5 |
| Saposnik G, Caplan LR, Gonzalez LA, Baird A, Dashe J, Luraschi A, Llinas R, Lepera S, Linfante I, Chaves C, Kanis K, Sica RE, Rey RC. Differences in stroke subtypes among natives and caucasians in Boston and Buenos Aires. Stroke 2000 October;31(10):2385-9 |
| Simonetti GB, Mono ML, Huynh-Do U, Michel P, Odier C, et al. Risk factors, aetiology and outcome of ischaemic stroke in young adults: the Swiss Young Stroke Study (SYSS) J Neurol. 2015 Sep;262(9):2025-32 |
| Sharma VK, Tsivgoulis G, Teoh HL, Ong BKC, Chan BPL. Stroke Risk Factors and Outcomes Among Various Asian Ethnic Groups in Singapore. J Stroke Cerebrovasc Dis. 2012 May;21(4):299-304. |
| Tan KS, Tan CT, Churilov L, Mackay M, Donnan GA. Ischaemic stroke in young adults: A comparative study between Malaysia and Australia. Neurology Asia 2010; 15(1) : 1 –9 |
| Tsong-Hai L, Wen-Chuin H, Chi-Jen C, Sien-Tsong C. Etiologic study of young ischemic stroke in Taiwan. Stroke 2002; 33: 1950–1955. |
| Uchino K, Risser JM, Smith MA, Moye LA, Morgenstern LB. Ischemic stroke subtypes among Mexican Americans and non-Hispanic whites: the BASIC Project. Neurology 2004 August 10;63(3):574-6 |
| Vallejos J, Jaramillo A, Reyes A, Illanes S, Orellana P, Manterola J, Diaz V. Prognosis of cryptogenic ischemic stroke: a prospective single-center study in Chile. J Stroke Cerebrovasc Dis 2012 November;21(8):621-8. |
| Varona JF, Guerra JM, Bermejo F, Molina JA, Gomez de laCamara A (2007) Causes of ischemic stroke in young adults, and evolution of the etiological diagnosis over the long term. EurNeurol 57:212–218 |
| Williams LS, Garg BP, Cohen M, Fleck JD, Biller J. Subtypes of ischemic stroke in children and young adults. Neurology 1997; 49: 1541–1545. |
| Wolf ME, Sauer T , Alonso A, Hennerici MG. Comparison of the new ASCO classification with the TOAST classification in a population with acute ischemic stroke. J Neurol (2012) 259:1284–1289 |
| Wu CY, Wu HM, Lee JD, Weng HH. Stroke risk factors and subtypes in different age groups: a hospital-based study. Neurol India 2010 November;58(6):863-8. |
| Cerrato P, Grasso M, Imperiale D, Priano L, Baima C, Giraudo M, Rizzuto A, Azzaro C, Lentini A, Bergamasco B. Stroke in Young Patients: Etiopathogenesis and Risk Factors in Different Age Classes. Cerebrovasc Dis 2004;18:154–159 |
| Chan MT, Nadareishvili ZG, Norris JW. Diagnosticstrategies in young patients with ischemic stroke incanada. Can J Neurol Sci 2000; 27:120-4. |
| Deleu D, Inshasi J, Akhtar N, Ali J, Vurgese T, Ali S, Rajan M, AlMutairy M, Zayed A, Paulose G, Nouri K, Thussu A, Miyares FR, Abdeen T, AlHail H, Alshubaili A, Mahmoud H. Risk factors, management and outcome of subtypes of ischemic stroke: a stroke registry from the Arabian Gulf. J Neurol Sci 2011 January 15;300(1-2):142-7 |
